# The impact of social isolation and perceived loneliness in older adults: a cross-sectional descriptive Chilean study

**DOI:** 10.3389/fpsyg.2025.1683933

**Published:** 2026-01-02

**Authors:** Christine Gierke, Carlos F. Navarro, Melissa Martinez, Carolina Delgado, Walthers Diaz-Gierke, Rocio Diaz, Gonzalo Farias

**Affiliations:** 1 Hospital Clínico Universidad de Chile, Servicio de Neurología y Neurocirugía, Santiago, Chile; 2 Social Isolation Research Center (SIRC), NiBG Instituto de Ciencias Biomedicas, Facultad de Medicina Universidad de Chile, Santiago, Chile; 3 Departamento de Neurociencias, Facultad de Medicina, Universidad de Chile, Santiago, Chile; 4 Escuela de Psicología, Universidad Adolfo Ibañez, Santiago, Chile; 5 Escuela de Psicología, FACSO Universidad de Chile, Santiago, Chile; 6 Centro de Investigacion de Clinica Avanzada (CICA), Facultad de Medicina, Universidad de Chile, Santiago, Chile

**Keywords:** social isolation, loneliness, older adults, depression, pandemic-related concerns

## Abstract

*Social Isolation* is defined as having minimal contact with other people, whether they are family or friends. At the same time, *Loneliness* is the subjective feeling of having less affection and closeness than desired in the intimate or relational sphere and of experiencing little proximity to family or friends. This study explores the psychological effects of the COVID-19 pandemic on elderly Chileans, focusing on the role of *Social Isolation*, *Loneliness,* and pandemic-related concerns and their impact on depressive symptoms. With 150 participants, this cross-sectional descriptive study revealed significant relationships between *SI*, *L*, depressive symptoms, and sex differences in their experiences. Despite no longer being in a restrictive phase of the pandemic, the findings highlight the interconnectedness of these factors and their ongoing impact on the mental health of the elderly population.

## 1 Introduction

### 1.1 Definitions and conceptual distinctions

Social isolation and loneliness represent distinct but interconnected psychosocial phenomena affecting older adults globally. Social isolation refers to the objective absence of social contacts and interactions, characterized by minimal engagement with family, friends, or community networks (Freedman and Nicolle, 2020). In contrast, loneliness constitutes the subjective experience of perceived social disconnection—the distressing feeling that one’s social relationships are insufficient in quantity or quality to meet emotional and social needs (Donovan and Blazer, 2020).

These constructs, while related, can exist independently. An individual may experience loneliness despite being surrounded by others if relationships lack emotional depth or meaning. Conversely, someone with limited social contact may not feel lonely if their few relationships are deeply satisfying and meaningful.

### 1.2 Physiological and psychological consequences

Loneliness transcends the emotional dimension and is associated with measurable physiological effects, including activation of stress response systems, elevated cortisol levels, and disrupted emotional regulation processes (Spreng et al., 2020). These biological changes contribute to increased vulnerability to anxiety and depression. Chronic loneliness correlates with reduced gray matter volumes in brain regions critical for emotional processing and memory, including the amygdala and hippocampus.

Social isolation similarly affects neurobiological functioning. Prolonged isolation reduces cognitive stimulation, potentially weakening brain structures associated with executive function and memory consolidation. Both conditions disrupt dopamine pathways, diminishing motivation and reward processing capabilities (Vitale and Smith, 2022).

The health consequences extend beyond mental health. Both loneliness and social isolation are associated with increased risk of cardiovascular disease, compromised immune function, cognitive decline, and premature mortality—effects comparable to established risk factors such as smoking and obesity (Holt-Lunstad, 2018).

### 1.3 Impact on older adults and global context

Older adults face vulnerability to social isolation and loneliness due to age-related transitions including retirement, health decline, mobility limitations, and bereavement. These life changes can disrupt established social networks and reduce opportunities for meaningful social engagement (Cacioppo and Patrick, 2008).

Recent research identifies social isolation as a modifiable contributor to dementia risk, emphasizing the critical importance of social connection for cognitive health in aging populations (Livingston et al., 2020–2024). The bidirectional relationship between loneliness and depression is particularly relevant for older adults, as loneliness can both precipitate and result from depressive episodes.

Gender differences in the experience of loneliness and social isolation among older adults are well-documented internationally. Women typically report higher levels of loneliness despite often maintaining larger social networks, possibly reflecting different expectations for social connection quality and emotional expression patterns (Baum et al., 2021).

### 1.4 Quality of life and healthy aging framework

Healthy aging encompasses multiple dimensions beyond the absence of disease, including quality of life, emotional well-being, social participation, and maintenance of functional independence. Social connections are fundamental to each of these dimensions. Research demonstrates that social participation and meaningful daily activities are essential components of active aging, contributing to physical health, cognitive vitality, and psychological well-being (Parra-Rizo et al., 2022).

The incorporation of loneliness as a social determinant of health into healthcare curricula represents an urgent need. Such inclusion would enhance healthcare professionals’ competencies in detecting and managing loneliness among older adults, optimize healthcare resource utilization, reduce clinical complications, and improve quality of life for this growing population (Parra-Rizo et al., 2025).

### 1.5 COVID-19 pandemic impact and Chilean context

The COVID-19 pandemic intensified existing challenges related to social isolation and loneliness, particularly among older adults who faced heightened health risks and prolonged social distancing measures (Heape, 2021). Chile, with 18.1% of its population aged 60 years or older (MINSAL, 2021), experienced significant increases in mental health concerns during the pandemic period.

Chilean older adults face unique contextual factors that may influence social isolation and loneliness experiences. The country’s rapid demographic transition, combined with urbanization and changing family structures, has altered traditional intergenerational support systems. Approximately 13.75% of Chilean older adults live alone (CASEN, 2017-2022), a proportion that may have increased during the pandemic.

Depression rates among Chilean adults over 60 reached 38% during the pandemic, compared to 24% pre-pandemic, with higher prevalence among women (CEVE-UC, 2020-2021-2022). These statistics underscore the urgent need for understanding psychosocial factors affecting this population’s mental health.

### 1.6 Latin American context and research gaps

Limited research exists on social isolation and loneliness among older adults in Latin American contexts, despite shared cultural characteristics that may influence these experiences differently than in Anglo-Saxon populations where most research has been conducted. Latin American cultures traditionally emphasize strong family bonds, intergenerational living arrangements, and community solidarity—factors that may provide protective effects against social isolation and loneliness.

However, rapid urbanization, economic migration, and changing social structures may be eroding these traditional protective factors. Understanding how Chilean and broader Latin American older adults experience social isolation and loneliness in contemporary contexts is essential for developing culturally appropriate interventions and policies.

### 1.7 Knowledge gaps and study rationale

Several critical knowledge gaps remain regarding social isolation and loneliness among Chilean older adults:

1. Prevalence and Distribution: Limited data exist on the prevalence of social isolation and loneliness among Chilean older adults, particularly in post-pandemic contexts.
2. Gender Differences: While international research documents gender differences in loneliness experiences, these patterns may differ in Chilean cultural contexts where traditional gender roles and family expectations may influence social connection patterns differently.
3. Cultural Factors: The role of Chilean-specific cultural factors—including family structures, social norms, and community resources—in moderating relationships between social isolation, loneliness, and mental health outcomes remains understudied.
4. Post-Pandemic Effects: The longer-term psychological effects of pandemic-related social restrictions on Chilean older adults require investigation, particularly regarding persistent concerns and their relationship to social connectedness.

### 1.8 Study objectives and hypotheses

In this context, the present study seeks to characterize post-pandemic levels of social isolation, loneliness, pandemic-related concerns, and depressive symptoms among Chilean older adults. Specific objectives include:

1. To determine the prevalence of social isolation, loneliness, and depressive symptoms in a sample of Chilean older adults.
2. To examine associations between these psychosocial variables.
3. To explore potential gender differences in these relationships.
4. To investigate the role of ongoing pandemic-related concerns in relation to social isolation, loneliness, and mental health.

Based on international literature and theoretical frameworks, it is hypothesized that:

- Social isolation and loneliness will be moderately correlated but represent distinct constructs.
- Both social isolation and loneliness will show significant positive associations with depressive symptoms.
- Gender differences will emerge, with women showing stronger associations between loneliness and depression.
- Pandemic-related concerns may show complex relationships with other variables, potentially serving both risk and protective functions.

## 2 Materials and methods

### 2.1 Study design

A descriptive cross-sectional study was conducted to provide a comprehensive snapshot of psychosocial conditions experienced by Chilean older adults in the post-pandemic period. This design was selected as the most appropriated approach for characterizing the prevalence and associations between social isolation, loneliness, pandemic-related concerns, and depressive symptoms, while acknowledging its limitation in establishing causal relationships.

Data collection occurred between March 2023 and December 2023 at the University of Chile’s Clinical Hospital as part of the AudioBrain Research Project.

#### 2.1.1 Participants and sampling limitations

The research employed a convenience sample of 150 Chilean participants [102 women (68%) and 48 men (32%)] recruited from Recoleta, Independencia, and Santiago municipalities. This sampling approach presents several critical limitations that significantly affect the generalizability of findings.

First, the substantial gender imbalance (68% women vs. 32% men) deviates markedly from expected gender distribution in the Chilean older adult population (approximately 55% women to 45% men: INE, 2021). This overrepresentation of women may bias sex-stratified analyses by:

- Inflating statistical power for detecting effects in women while reducing power for men.
- Potentially overestimating prevalence of conditions more common in women.
- Limiting external validity of findings related to gender differences.

Second, geographic restriction to three urban municipalities within the Santiago Metropolitan Region severely limits generalizability to the broader Chilean older adult population. Chile exhibits substantial geographic, socioeconomic, and cultural heterogeneity across its regions. Rural populations, which comprise approximately 12% of Chilean older adults (CASEN, 2017-2022), may experience fundamentally different patterns of social isolation and loneliness due to:

- Different family structure patterns and intergenerational living arrangements.
- Varying access to healthcare and social services.
- Distinct cultural norms regarding aging and social support.
- Economic factors that may differentially affect mental health outcomes.

Third, recruitment through a clinical hospital setting introduces systematic selection bias. Participants accessing healthcare services may differ significantly from community-dwelling older adults who do not regularly utilize medical services. This hospital-based recruitment may have resulted in:

- Overrepresentation of individuals with higher health consciousness or greater health concerns.
- Potential oversampling of those with better healthcare access and insurance coverage.
- Exclusion of homebound or institutionalized older adults.
- Bias toward participants with sufficient mobility and cognitive capacity to attend hospital appointments.

All participants signed informed consent and were assessed at the University of Chile’s Clinical Hospital.

#### 2.1.2 Sample size and power analysis limitations

No formal sample size calculation or statistical power analysis was conducted *a priori*, representing a methodological limitation. The total sample size (*n* = 150) provides adequate power (>0.80) for detecting medium effect sizes (r ≥ 0.30) in the full sample, but gender-stratified analysis are underpowered, particularly for men (*n* = 48). This limitation affects:

- The reliability of null findings, particularly for men.
- The precision of correlation estimates in smaller subgroups.
- The ability to detect clinically meaningful but statistically modest associations.
- The robustness of comparisons between gender groups.

#### 2.1.3 Implications for interpretation and generalizability

These sampling limitations have several critical implications for the interpretation and generalizability of findings:

1. *Limited External Validity*: Findings may not generalize to rural Chilean populations, other geographic regions, or community-dwelling older adults who do not access healthcare services regularly.
2. *Biased Prevalence Estimates*: The reported prevalence rates of social isolation (42%), loneliness (26%), and depression (30%) may not accurately reflect population-level prevalence in Chilean older adults.
3. *Compromised Gender Comparisons*: Sex-stratified analyses should be interpreted with extreme caution due to the unbalanced sample and differential statistical power between groups.
4. *Selection Effects*: The volunteer nature of participation and hospital-based recruitment may have selected for individuals with particular characteristics (higher health engagement, better functional status) that could systematically differ from the broader population.

These limitations underscore the necessity for replication studies using probability sampling methods, balanced gender representation, and inclusion of diverse geographic and socioeconomic populations to establish the broader applicability of these findings to Chilean older adults.

#### 2.1.4 Inclusion and exclusion criteria

Inclusion criteria: Adults aged 60 years or older, community-dwelling, Spanish-speaking, capable of providing informed consent.

Exclusion criteria: Severe cognitive impairment preventing informed consent, hospitalized individuals, inability to complete assessment protocols.

### 2.2 Instruments

All instruments were administered face-to-face by trained clinical psychologists in a controlled hospital environment to ensure standardized conditions and immediate clarification of participant queries.

#### 2.2.1 Steptoe social isolation index

This 5-point scale measures objective *Social Isolation,* with scores ≥2 indicating presence of social isolation. The instrument demonstrates good internal consistency (Cronbach’s *α* = 0.80) internationally. *Critical Limitation*: This scale has not been validated in Chilean populations. Cultural differences in social network conceptualization and family structure may affect measurement accuracy and interpretation (Steptoe et al., 2015).

#### 2.2.2 UCLA three-item loneliness scale

This brief scale assesses subjective loneliness through three questions, with scores ≥5 indicating high loneliness levels. International reliability is good (Cronbach’s α = 0.83). *Critical Limitation*: No Chilean validation exists. The cultural expression and interpretation of loneliness may differ significantly in Chilean contexts compared to populations where this instrument was developed (Hugues et al., 2004).

#### 2.2.3 Yesavage’s geriatric depression scale (GDS-15)

This 15-item dichotomous scale measures depressive symptoms in older adults. Cutoff scores: 0–4 (normal), 5–8 (mild depression), 9–11 (moderate depression), 12–15 (severe depression). Internal consistency is good (Cronbach’s α = 0.84). This scale has Chilean validation (Sheikh and Yesage, 1986).

#### 2.2.4 Pandemic-related concern scale (*ad hoc*)

A single-item Likert scale (1–10) was developed specifically for this study to assess ongoing COVID-19-related concerns, given the absence of validated instruments measuring pandemic-specific worries in elderly populations. Scores ≥5 indicate significant concern. *Critical Limitation*: This scale lacks psychometric validation, and results should be interpreted with extreme caution.

### 2.3 Instrument validation limitations

A critical limitation of this study is the use of instruments that have not been specifically validated for the Chilean older adult population. The Steptoe Social Isolation Index and UCLA-3 Loneliness Scale, while demonstrating robust psychometric properties internationally, lack formal validation in Chilean cultural contexts. This limitation was unavoidable due to the absence of validated Chilean alternatives and the urgent need to assess these constructs during the post-pandemic period.

Similar methodological approaches have been employed in Latin American research contexts. Studies in Mexico (García-Peña et al., 2022), Colombia (Restrepo et al., 2022), and Argentina (Fernández-Ballesteros et al., 2020) have utilized internationally developed scales for loneliness and social isolation assessment in the absence of locally validated instruments. The pandemic-related concern scale was developed *ad hoc* for this study due to the complete absence of validated instruments measuring COVID-19-specific concerns in elderly populations globally.

Future research should prioritize the cultural adaptation and validation of these instruments for Chilean populations, considering linguistic nuances, cultural expressions of social connection, and region-specific interpretations of loneliness and isolation.

#### 2.3.1 Cultural adaptation and translation issues

The use of non-validated instruments represents a fundamental methodological limitation. Spanish versions of international scales were used without formal cultural adaptation processes. Future research should prioritize validation of these instruments in Chilean populations, considering linguistic nuances and cultural expressions of social connection.

## 3 Procedure

Assessments were conducted individually in private rooms at CIDIN facilities, at the University of Chile’s Clinical Hospital. Each session lasted approximately 10–15 min. Standardized administration protocols were followed to minimize examiner bias. Participants were informed they could withdraw at any time without consequences.

## 4 Ethical considerations

This study adhered to Declaration of Helsinki principles. Ethical approval was obtained from the Scientific Research Ethics Committee of the University of Chile’s Clinical Hospital (record no. 71, October 20, 2020).

Informed consent capacity was evaluated for all participants. Participants with elevated depressive symptoms (GDS-15 ≥ 9) were provided with mental health resource information and referral options. All personal data were treated with strict confidentiality and used exclusively for research purposes.

## 5 Statistical analysis

Data normality was assessed using Shapiro–Wilk tests, revealing non-normal distributions (*p* < 0.05) for most variables. Consequently, non-parametric statistical methods were employed throughout.

Spearman’s rank correlation coefficients were calculated instead of Pearson correlations due to the non-normal data distribution. This non-parametric approach provides more robust estimates of association strength for the observed data characteristics.

For categorical variables, Chi-square tests were used to compare proportions between groups. For continuous variables, Mann–Whitney U tests were employed to compare distributions between men and women.

Three separate correlation matrices were constructed: total sample, men only, and women only. The significance level was set at α = 0.05. No adjustment for multiple comparisons was applied, which should be considered when interpreting results.

**Figure 1:**
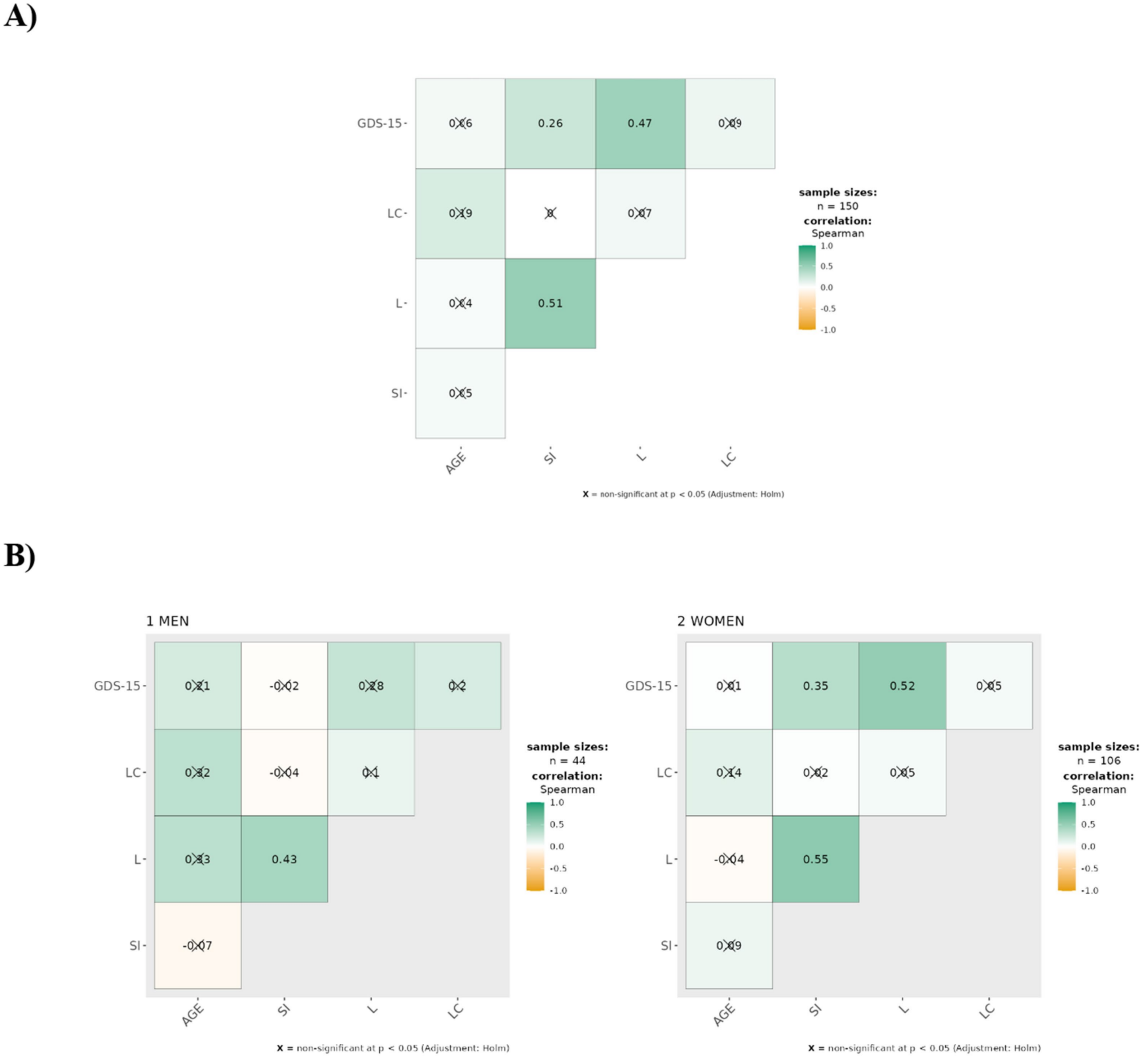
Spearman correlation matrices between Social Isolation (SI), Loneliness (L), Depression (GDS-15), Age and Pandemic-Related Concern (LC), by sex. **(A)** Shows the total sample (*n* = 150), **(B)** Corresponds to men (*n* = 48), and women (*n* = 102). Correlation coefficients (r) were calculated using Spearman method. Very strong positive (≥0.70), Strong positive (0.50–0.69), Moderate positive (0.30–0.49), Weak positive (0.10–0.29), non-significant (<0.10 or *p* ≥ 0.05), Weak negative (−0.10—0.29), Moderate negative (−0.30 to −0.49).

All analyses were conducted using Jamovi 2.7 Software. Effect sizes and 95% confidence intervals were calculated for all correlation coefficients using Fisher’s z-transformation method (Table 1; Figure 1).

**Table 1:** Spearman correlation (*r*) coefficients between key variables by group.

Correlation	Total (*n* = 150)	Men (*n* = 48)	Women (*n* = 102)
SI – L	*r* = 0.513*** (*p* < 0.001)	*r* = 0.487*** (*p* < 0.001)	*r* = 0.509*** (*p* < 0.001)
SI – GDS-15	*r* = 0.258** (*p* = 008)	*r* = −0.0174 (*p* = 0.921)	*r* = 0.267** (*p* = 0.004)
SI – LC	*r* = −0.056 (*p* = 0.496)	*r* = −0.098 (*p* = 0.503)	*r* = −0.043 (*p* = 0.668)
SI – AGE	*r* = 0.065 (*p* = 0.426)	*r* = 0.055 (*p* = 0.712)	*r* = 0.0781 (*p* = 0.471)
L – GDS-15	*r* = 0.468*** (*p* < 0.001)	*r* = 0.223* (*p* = 0.126)	*r* = 0.451*** (*p* < 0.001)
L – LC	*r* = 0.028 (*p* = 0.734)	*r* = 0.056 (p = 0.702)	*r* = 0.003 (*p* = 0.975)
L – AGE	*r* = 0.037 (*p* = 0.769)	*r* = 0.321** (*p* = 0.025)	*r* = −0.045 (*p* = 0.679)
GDS-15 – LC	*r* = 0.058 (*p* = 0.479)	*r* = 0.115 (*p* = 0.433)	*r* = 0.021 (*p* = 0.834)
GDS-15 – AGE	*r* = 0.055 (*p* = 0.915)	*r* = 0.139 (*p* = 0.342)	*r* = −0.031 (*p* = 0.753)
LC – AGE	*r* = 0.188* (*p* = 0.008)	*r* = 0.456*** (*p* < 0.001)	*r* = 0.106 (*p* = 0.284)

*p* = Spearman’s rho coefficient. *p < 0.0.05, **p < 0.01, ***p < 0.001. 95% confidence intervals should be calculated for final publication.

## 6 Results

The database included 150 individuals (± 69.5, SD = 5.93). Of these, 68% were women (*n* = 102, ±73.5, SD = 5.99) and 32% were men (*n* = 48, ±72.5, SD = 5.79). Their Social Priority Index (SPI) is an indicator integrating relevant aspects of communal social development across three dimensions (income, health, and education), ranking all the municipalities by giving them a social priority rank and a numerical value (100 to 0, where a higher SPI indicates a worse socioeconomic condition). Recoleta is ranked 19^th^, Independencia 26^th^, and Santiago 41^st^ (SEREMI, 2022) educational level >8 years. Regarding marital status, 38% were married, 16% were separated, 10% were widowed and 36% were single, the results do not show statistical significance between men and women.

The social isolation index indicates that 42% of the participants (*n* = 63, M = 1.34, SD = 1.02, Mdn = 1.0) presented social isolation; 26% (*n* = 39, M = 4.02, SD = 1.53, Mdn = 3.0) reported experiencing loneliness. The results do not show significant differences in proportion and distribution between men and women in either *SI* (x^2^ = 0.825, *U* = 0.696) or L (x^2^ = 0.290, *U* = 0.412).

Regarding the Geriatric Depression Scale index (GDS-15), 30% of the sample (*n* = 44) showed indicators of depression (x^2^ = 0.470, *U* = 0.848). Additionally, the pandemic-related concern Likert-scale revealed that 91% of the sample (*n* = 137) reported high levels of concern (>5) (x^2^ = 0.127, *U* = 1.0). The results do not show significant differences in proportion and distribution between men and women (Table 2).

**Table 2:** Descriptive data.

Variable	Sex	N	Mean	Median	SD	Min	Max	Asymmetry	SE	Kurtosis	SE	SW	p
SI	Men	48	1.29	1.0	0.944	0	4	0.478	0.343	0.1755	0.674	0.882	<0.001
SI	Women	102	1.36	1.0	1.07	0	5	0.613	0.239	0.1832	0.474	0.886	<0.001
L	Men	48	3.71	3.0	1.031	3	7	1.356	0.343	1.1319	0.674	0.717	<0.001
L	Women	102	4.17	3.0	1.718	3	9	1.552	0.239	1.3883	0.474	0.71	<0.001
GDS-15	Men	48	2.92	2.0	2.82	0	13	1.669	0.343	3.3924	0.674	0.839	<0.001
GDS-15	Women	102	3.52	2.5	3.308	0	13	0.899	0.239	−0.0892	0.474	0.887	<0.001
LC	Men	48	7.33	8.0	2.215	3	10	−0.344	0.343	−1.0152	0.674	0.909	0.001
LC	Women	102	7.05	7.0	2.168	1	10	−0.469	0.239	−0.1196	0.474	0.937	<0.001
AGE	Men	48	70.17	69.0	5.788	61	84	0.307	0.343	−0.7567	0.674	0.964	0.148
AGE	Women	102	69.25	69.0	5.994	60	87	0.552	0.239	0.1722	0.474	0.961	0.005

Descriptive statistics for each variable by sex. (SD) Standard Deviation. (SE) Standard error. Shapiro Wilk (SW).

### 6.1 Correlation between social isolation, perception of loneliness, pandemic-related concern and depressive symptoms

Moderate positive correlations were observed between social isolation and loneliness across all groups: total sample (*ρ* = 0.498, *p* < 0.001), men (*ρ* = 0.487, *p* < 0.001), and women (*ρ* = 0.509, *p* < 0.001). These statistically significant associations indicate that individuals experiencing higher levels of objective social isolation tended to also report greater subjective loneliness, though the moderate strength suggests these remain distinct constructs. The correlation was slightly stronger among women compared to men, though this difference was not statistically tested.

The relationship between social isolation and depressive symptoms showed a weak positive correlation in the total sample (*ρ* = 0.203, *p* = 0.008) and women (ρ = 0.267, *p* = 0.004) but was non-significant in men (*ρ* = 0.014, *p* = 0.921). This gender difference may reflect true variation in how social isolation relates to depression between sexes, though the smaller male subsample (*n* = 48) limits statistical power for detecting associations in this group.

Loneliness demonstrated moderate positive correlations with depressive symptoms in the total sample (*ρ* = 0.389, *p* < 0.001) and women (*ρ* = 0.451, *p* < 0.001), indicating that higher loneliness was associated with greater depressive symptomatology. In men, the correlation was weaker and did not reach statistical significance (*ρ* = 0.223, *p* = 0.126), though this may reflect insufficient power rather than absence of association.

Weak, non-significant correlations were observed between social isolation and pandemic-related concern across all groups (total sample: *ρ* = −0.056, *p* = 0.496; women: *ρ* = −0.043, *p* = 0.668; men: *ρ* = −0.098, *p* = 0.503). These associations do not reach statistical significance and should be interpreted cautiously. The lack of significant associations suggests that pandemic-related concern may not be directly related to social isolation levels in this sample, though larger studies would be needed to confirm this finding.

Similarly, loneliness showed no significant associations with pandemic-related concern in any group (total: *ρ* = 0.028, *p* = 0.734; women: *ρ* = 0.003, *p* = 0.975; men: *ρ* = 0.056, *p* = 0.702). Depression also showed no meaningful correlation with pandemic concern across groups.

A weak, non-significant correlation was observed between loneliness and age in women (*ρ* = −0.041, *p* = 0.679), indicating no meaningful association between these variables in this sample. Interestingly, men showed a weak positive correlation between loneliness and age (*ρ* = 0.321, *p* = 0.025), suggesting that older men in the sample tended to report slightly higher loneliness levels, though this finding requires replication.

The most notable finding regarding age was its moderate positive correlation with pandemic-related concern among men (*ρ* = 0.456, *p* < 0.001), indicating that older men expressed significantly greater pandemic-related worries. This association was weaker and non-significant in women (*ρ* = 0.106, *p* = 0.284), suggesting potential gender differences in how age relates to pandemic concerns (Table 1; Figure 1).

## 7 Discussion

This cross-sectional study examined relationships between social isolation, loneliness, pandemic-related concerns, and depressive symptoms among 150 Chilean older adults during the post-pandemic period. While findings provide preliminary insights into these relationships within a Chilean context, substantial methodological limitations significantly constrain interpretation and generalizability.

### 7.1 Summary of main findings and comparison with previous literature

#### 7.1.1 Prevalence findings

The study documented concerning prevalence rates: 42% presented social isolation, 26% reported loneliness, 30% exhibited depressive symptoms, and 91% expressed high levels of pandemic-related concern. However, these estimates require cautious interpretation given the convenience sampling method and hospital-based recruitment, which may not reflect population-level prevalence among Chilean older adults.

The depression prevalence aligns with reported increases during Chile’s pandemic period (38% among adults over 60; CEVE-UC, 2020-2021-2022), though direct comparisons are limited by methodological differences. The high rate of persistent pandemic concern (91%) 2 years post-pandemic onset suggests enduring psychological impacts among this population.

#### 7.1.2 Association patterns

Moderate positive correlations were observed between social isolation and loneliness across all groups: total sample (*ρ* = 0.498, *p* < 0.001), men (*ρ* = 0.487, *p* < 0.001), and women (*ρ* = 0.509, *p* < 0.001), with slightly stronger associations in women, supports theoretical distinctions between objective social disconnection and subjective loneliness established in international literature (Donovan and Blazer, 2020). This finding aligns with recent systematic reviews demonstrating that while related, these constructs have independent effects on mental health outcomes (Holt-Lunstad, 2024).

The association between loneliness and depressive symptoms was stronger in women (*ρ* = 0.451, *p* < 0.001) compared to men (*ρ* = 0.223, *p* = 0.126), though the non-significant finding in men may reflect insufficient statistical power due to the unbalanced sample. This pattern is consistent with international research documenting gender differences in loneliness manifestation, though sampling limitations prevent definitive conclusions about sex differences in Chilean contexts.

Similarly, social isolation showed weak but significant positive associations with depressive symptoms in the total sample (*ρ* = 0.203, *p* = 0.008) and women (*ρ* = 0.267, *p* = 0.004), but no association in men (*ρ* = 0.014, *p* = 0.921). This pattern further suggests gender differences in how objective social disconnection relates to mental health outcomes, though power limitations in the male subsample prevent definitive conclusions.

#### 7.1.3 Latin American context

Limited research exists on social isolation and loneliness in Latin American older adult populations, making regional comparisons challenging. A recent Brazilian study reported similar depression rates (32%) among community-dwelling older adults during the pandemic period. Research from Mexico highlighted protective effects of extended family networks against pandemic-related mental health impacts, suggesting cultural factors may moderate these relationships in Latin American contexts (García-Peña et al., 2022).

The study referenced by reviewers provides valuable regional context, demonstrating comparable patterns of social isolation and mental health outcomes across different Latin American countries, though methodological differences limit direct comparison with the present findings (Kammar-García et al., 2023).

Notably, pandemic-related concern showed minimal associations with other study variables, suggesting that specific COVID-19 worries may represent a distinct construct from more general patterns of social isolation and loneliness. This contrasts with some literature suggesting stronger relationships between pandemic-specific concerns and mental health outcomes, though differences in assessment timing (2 years post-pandemic onset) and measurement approaches may account for these discrepancies.

Besides that, age showed different patterns by gender. Among men, older age was significantly associated with greater loneliness (*ρ* = 0.321, *p* = 0.025) and substantially higher pandemic-related concern (*ρ* = 0.456, *p* < 0.001). These associations were weak and non-significant among women, suggesting potential gender differences in how aging relates to social disconnection and health-related worries in Chilean contexts.

### 7.2 Theoretical explanations and underlying mechanisms

#### 7.2.1 Neurobiological mechanisms

The observed associations between social isolation, loneliness, and depression can be understood through multiple theoretical frameworks. From a neurobiological perspective, chronic loneliness activates hypothalamic–pituitary–adrenal axis responses, leading to elevated cortisol levels and inflammatory processes that may contribute to depressive symptomatology (Spreng et al., 2020). Loneliness has been associated with structural and functional alterations in brain regions involved in emotional regulation, including the amygdala and prefrontal cortex, which may underlie increased vulnerability to mood disorders.

#### 7.2.2 Psychosocial pathways

From psychosocial perspectives, loneliness may contribute to negative cognitive patterns characteristic of depression, including rumination, hopelessness, and negative self-evaluation. Recent network analysis demonstrates that rumination specifically about loneliness modulates the relationship between loneliness and depression, with this cognitive process playing a critical mediating role (Luo et al., 2025). The bidirectional relationship between loneliness and depression suggests that interventions targeting either construct may have beneficial effects on both, though the cross-sectional design prevents causal inferences. Longitudinal evidence indicates that higher loneliness levels predict greater depression severity over time, supporting the potential value of loneliness-reduction interventions for depression prevention in older adults (Alonzo et al., 2020).

Carstensen’s Socioemotional Selectivity Theory provides additional theoretical context for understanding age-related changes in social relationships. According to this framework, older adults increasingly prioritize emotionally meaningful relationships over larger social networks, which may influence both the experience of loneliness and its relationship to mental health outcomes (Carstensen, 2021).

#### 7.2.3 Gender differences and cultural factors

The observed gender differences in the associations between loneliness and depression may reflect multiple interconnected factors. Women typically maintain larger and more diverse social networks compared to men but may experience greater psychological distress when these relationships are perceived as inadequate or emotionally unsatisfying (Barreto et al., 2025). Research indicates that women are particularly vulnerable to the psychological effects of loneliness, reporting higher levels of depression and anxiety related to social isolation compared to men, even when controlling for objective social network size (Morales Chaine et al., 2021).

Additionally, gender socialization patterns may influence how loneliness is experienced and expressed. Women may be more likely to recognize, label, and report feelings of loneliness and depression due to greater emotional awareness and lower stigma around discussing emotional distress. These reporting differences, combined with genuine variations in how social disconnection affects mental health, potentially contribute to the stronger observed associations between loneliness and depressive symptoms among women in this sample.

Cultural factors specific to Chilean and Latin American contexts may further modulate these gender differences. Strong family orientation and intergenerational support systems characteristic of Latin American cultures may provide protective effects against social isolation, though rapid urbanization and changing family structures may be attenuating these traditional buffers. The role of traditional gender roles in Chilean society—where women often serve as primary caregivers and relationship maintainers—may contribute to different experiences of social connection and its disruption. When women experience isolation or perceive their social relationships as inadequate, this may represent a more fundamental disruption of their socially defined roles, potentially amplifying distress.

The lack of significant associations between social isolation and depression among men in this sample (*ρ* = 0.014, *p* = 0.921) requires cautious interpretation and may reflect several factors. First, the smaller male subsample size (*n* = 48) compared to women (*n* = 102) substantially reduces statistical power to detect associations, even if they exist. Power analysis suggests that with this sample size, only moderate to large effects would be reliably detected in men.

Second, men may express psychological distress from social disconnection through alternative pathways not fully captured by traditional depression screening instruments. Research indicates that men are more likely to manifest distress through externalized symptoms such as irritability, anger, substance use, or risk-taking behaviors rather than the internalized symptoms (sadness, crying, hopelessness) emphasized in depression scales (Seidler et al., 2021). Additionally, masculine gender norms may discourage men from recognizing or reporting emotional symptoms associated with isolation, contributing to underestimation of mental health impacts (Pirkis et al., 2023).

Third, older men who do access healthcare services—as in this hospital-recruited sample—may represent a select group with better help-seeking behaviors and social support than community-dwelling men who avoid healthcare engagement. This selection bias may have further attenuated observable associations between isolation and depression among male participants.

#### 7.2.4 Pandemic-specific considerations

The minimal associations observed between pandemic-related concern and most study variables (social isolation, loneliness, and depression) require careful interpretation. Two years post-pandemic onset, specific COVID-19 worries may have evolved into more generalized anxiety patterns or become integrated into broader health concerns. Research indicates that while acute pandemic-related anxiety was prevalent early in the pandemic, particularly among older adults, these concerns have shifted over time as populations adapted to the endemic phase of COVID-19 (Kadowaki and Wister, 2022).

However, a notable exception emerged: the strong positive association between age and pandemic-related concern among men (*ρ* = 0.456, *p* < 0.001), while women showed no such relationship (*ρ* = 0.106, *p* = 0.284). This gender-specific pattern suggests that older males may remain particularly vigilant about health threats. This heightened concern may reflect rational risk assessment, as men experienced substantially higher COVID-19 mortality rates globally. Studies documented that male death rates from COVID-19 were approximately 1.6 times higher than female rates, with the gender gap widest in middle and older age groups (Reeves and Ford, 2022). In the United States alone, at least 65,000 more men than women died from COVID-19 by early 2022, with men accounting for approximately 57% of all COVID-19 deaths (Ramírez-Soto et al., 2021).

The persistent pandemic concern among older men in this Chilean sample, assessed 2 years post-pandemic onset, may therefore represent informed awareness of their elevated vulnerability rather than maladaptive anxiety. Alternatively, this pattern could reflect cohort-specific experiences during Chile’s pandemic response, where older adults faced prolonged restrictions and witnessed high mortality rates among their age group. The absence of similar age-pandemic concern associations among women suggests potential gender differences in how health threat information is processed or expressed, though further research is needed to clarify these mechanisms.

### 7.3 Clinical and policy implications

#### 7.3.1 Healthcare system integration

Given the methodological limitations of this study, clinical recommendations must be offered tentatively and require validation through implementation research. However, the documented prevalence of social isolation (42%) and loneliness (26%) among participants, combined with their associations with depressive symptoms, suggests potential opportunities for healthcare system intervention.

Screening for both objective social isolation and subjective loneliness in primary care settings may represent an important starting point for addressing these conditions, as these constructs demonstrated related but distinct patterns in this sample. Primary care providers are well-positioned to assess loneliness, as older adults visit primary care settings 5–6 times per year on average, with lonely individuals averaging one additional visit annually (Williams-Farrelly et al., 2024). However, currently no national-level recommendations exist for screening social isolation and loneliness (National Academies of Sciences, Engineering, and Medicine, 2020), highlighting the need for evidence-based screening protocols.

In the Chilean context, integration of loneliness and social isolation assessment into the existing CESFAM (Family Healthcare Centers) network could leverage established infrastructure for geriatric care. Chile’s primary healthcare system already includes comprehensive health assessments for older adults (Examen de Medicina Preventiva del Adulto Mayor - EMPAM), providing a potential framework for incorporating psychosocial screening. Training healthcare professionals to recognize and address social isolation and loneliness has been identified as a critical component of effective intervention (Freedman and Nicolle, 2020), though such training programs require cultural adaptation for Chilean healthcare contexts.

The stronger associations between loneliness and depression observed among women in this sample suggest potential value in gender-sensitive screening approaches, though the sampling limitations and underpowered male subsample preclude definitive recommendations. Research indicates that addressing loneliness in primary care is particularly relevant for older adults, as those experiencing loneliness utilize healthcare services more frequently (Williams-Farrelly et al., 2024), suggesting that identification and intervention could optimize resource utilization while improving patient outcomes.

Implementation research is needed to establish feasible, acceptable, and effective approaches for integrating loneliness screening into Chilean primary care workflows, considering resource constraints, healthcare provider workload, and cultural appropriateness of interventions.

#### 7.3.2 Community-based interventions

Evidence from systematic reviews and meta-analyses indicates that community-based interventions can effectively reduce loneliness among older adults, though effect sizes vary considerably depending on intervention type and implementation quality. A comprehensive meta-analysis of 35 randomized controlled trials found that the most effective approaches include cognitive engagement and restructuring interventions, social behavioral activation programs, and acceptance of aging interventions (Noone et al., 2024). Network meta-analysis suggests that psychological interventions are most effective for reducing late-life loneliness, though increasing social dynamics and connectivity may provide additional benefits (Zhao et al., 2023).

In the Chilean context, several community-based programs for older adults exist through SENAMA (Servicio Nacional del Adulto Mayor), though systematic evaluation of their effectiveness in reducing loneliness and social isolation remains limited. The “Vínculos” program, which promotes social connection among older adults, was adapted during the pandemic to “Televínculos” to maintain social support despite physical distancing measures (Fundación Chile, 2020). Research in Chilean contexts indicates that social participation and neighborhood social identification are associated with improved well-being and health outcomes among older adults (Varela et al., 2020), suggesting potential value in community-based approaches.

Culturally appropriate interventions that leverage traditional Latin American values of family connection and community solidarity while addressing contemporary challenges such as urbanization and changing family structures may be particularly relevant for Chilean populations, though this hypothesis requires empirical testing. Programs should consider integrating intergenerational activities, which align with cultural values of family cohesion and may reduce isolation while promoting reciprocal benefits across age groups.

Gender-specific considerations warrant attention in program design and implementation. Meta-analytic evidence suggests that intervention effectiveness may vary by participant characteristics, including gender (Noone et al., 2024), though few studies have systematically evaluated gender-specific approaches. Given the stronger associations between loneliness and depression observed among women in this sample, and the different patterns of social connection maintained by women and men, tailored intervention strategies may be warranted. Women may benefit more from interventions focused on relationship quality and emotional intimacy, while men may respond better to activity-based or task-oriented social programs, though these hypotheses require validation in Chilean contexts.

Effective interventions should address both objective social network enhancement (increasing frequency and range of social contacts) and subjective experiences of loneliness and social connection quality. The moderate correlation between social isolation and loneliness observed in this study (*ρ* = 0.498) suggests that while related, these constructs require distinct intervention approaches. Programs focusing solely on increasing social contacts without attending to relationship quality may fail to reduce subjective loneliness experiences.

Implementation research is critically needed to establish feasible, acceptable, and effective community-based interventions adapted to Chilean cultural contexts, healthcare infrastructure, and resource availability. Evaluation of existing SENAMA programs using standardized loneliness and social isolation measures would provide valuable evidence for optimizing these initiatives.

#### 7.3.3 Policy recommendations

Public policies addressing social isolation and loneliness among older adults require evidence-based approaches that consider both international best practices and Chilean-specific contextual factors. While this study’s methodological limitations preclude definitive policy prescriptions, the documented prevalence of these conditions and their associations with depressive symptoms suggest several policy directions worthy of consideration and further evaluation.

##### 7.3.3.1 Age-friendly cities and community infrastructure

Chile has participated in WHO’s Age-Friendly Cities initiative since the early 2000s, with municipalities such as Lampa implementing age-friendly practices including territorial meetings focused on services for older adults, transportation for social activities, and community celebrations that provide opportunities for social connection (Chung et al., 2021). Chile led the launch of the Decade of Healthy Ageing 2021–2030, demonstrating national commitment to improving conditions for older people (Gobierno de Chile, 2021). However, expansion of age-friendly infrastructure beyond pilot municipalities remains incomplete.

###### 7.3.3.1.1 Policy recommendations include

- Systematic implementation of WHO’s eight-domain age-friendly framework across Chilean municipalities, particularly in areas with rapidly aging populations.
- Development of accessible public spaces that facilitate intergenerational interaction and social participation.
- Integration of social connectedness considerations into urban planning and public transportation policies.

##### 7.3.3.2 Social prescribing programs

Social prescribing interventions, which link individuals to community-based activities and services to address social determinants of health, have demonstrated effectiveness in addressing social isolation and loneliness through 11 identified intervention types (Paque et al., 2023). Potential benefits for older adults include improving health and wellbeing, reducing social isolation and loneliness, increasing resilience, and potentially preventing hospital admissions (Ghogomu et al., 2024). Recent evidence from natural experiments during COVID-19 lockdowns demonstrated that community groups as social prescriptions significantly combat loneliness and health degeneration among aging populations during periods of isolation (GBD 2023 Demographics Collaborators, 2025).

###### 7.3.3.2.1 Implementation of social prescribing within Chilean primary healthcare would require

- Training of “link workers” or community health workers (potentially leveraging existing roles such as *técnicos paramédicos*) to connect older adults with community resources.
- Development of comprehensive directories of community-based organizations and activities suitable for older adults.
- Integration of social prescriptions into electronic health records to streamline processes and facilitate follow-up (Temitope, 2025).
- Evaluation frameworks to assess effectiveness and cost-effectiveness in Chilean contexts.

##### 7.3.3.3 SENAMA program enhancement and evaluation

SENAMA currently operates multiple programs targeting older adult wellbeing, including the “Vínculos” program promoting social connection and the “Buen Vivir” initiative. However, systematic evaluation of these programs’ effectiveness in reducing loneliness and social isolation using standardized measures remains limited. Policy priorities should include:

- Rigorous evaluation of existing SENAMA programs using validated loneliness and social isolation assessment tools.
- Expansion of evidence-based programs to underserved populations, including rural areas and low-income urban communities.
- Development of gender-sensitive program components, given the differential associations between loneliness and mental health outcomes observed among women and men.

##### 7.3.3.4 Mental health service integration

Social prescribing to mitigate negative downstream effects of loneliness should be considered a clinical and public health priority (Hough et al., 2023). Integration of loneliness screening and intervention into Chile’s mental health services requires:

- Training mental health professionals to assess and address social isolation and loneliness as risk factors for depression.
- Development of culturally appropriate group-based interventions that leverage Chilean values of family and community solidarity.
- Creation of referral pathways between mental health services and community-based social connection programs.

##### 7.3.3.5 Implementation challenges and research needs

Successful policy implementation faces several challenges in Chilean contexts:

- Resource constraints within the public healthcare system.
- Geographic barriers in rural and remote areas.
- Need for cultural adaptation of evidence-based interventions developed in other cultural contexts.
- Limited baseline data on population-level prevalence of loneliness and social isolation.

Critical research needs include:

- Population-representative prevalence studies using validated instruments.
- Implementation research evaluating feasibility, acceptability, and effectiveness of interventions in Chilean contexts.
- Cost-effectiveness analysis to inform resource allocation.
- Longitudinal studies establishing causal relationships and identifying optimal intervention timing.

These policy recommendations must be considered tentative given this study’s methodological limitations. Implementation should proceed cautiously with robust evaluation frameworks to establish effectiveness before widespread scaling.

### 7.4 Study limitations

This study has several critical limitations that significantly constrain the interpretation and generalizability of findings. These limitations must be carefully considered when evaluating the validity and applicability of results.

#### 7.4.1 Methodological and measurement limitations

##### 7.4.1.1 Instrument validation

The use of instruments not validated in Chilean populations represents a fundamental methodological limitation. The Steptoe Social Isolation Index and UCLA-3 Loneliness Scale were developed and validated in North American and European contexts, where cultural conceptualizations of social connection, family structures, and expressions of loneliness may differ substantially from Chilean experiences. Research demonstrates that loneliness is a culturally embedded construct, with significant variation across cultures in what constitutes adequate social connection and how loneliness is expressed and experienced (Barreto et al., 2021).

Specifically, Chilean cultural norms emphasizing extended family networks and intergenerational co-residence may influence both the experience of social isolation and its measurement. Items assessing frequency of contact with friends may have different meanings in cultures where family relationships predominate over friendship networks. Without local validation studies, the accuracy and cultural appropriateness of these measures in Chilean contexts remains uncertain.

The *ad hoc* pandemic concern scale lacks any psychometric validation, including established reliability, validity, or normative data. This single-item measure cannot capture the multidimensional nature of pandemic-related concerns (health worries, economic concerns, social restrictions impact) and provides no information about measurement error or stability over time.

##### 7.4.1.2 Study design limitations

The cross-sectional design fundamentally prevents causal inferences about relationships between variables. While associations were observed between loneliness and depression, the direction of causality cannot be determined. Depression may lead to withdrawal and subsequent loneliness, loneliness may contribute to depressive symptoms, or bidirectional relationships may exist. Additionally, unmeasured third variables (such as personality traits, chronic health conditions, or life events) may account for observed associations.

Temporal relationships and potential mediating or moderating factors cannot be examined with cross-sectional data. The assessment captured only a single time point, providing no information about the stability or change in social isolation, loneliness, or depressive symptoms over time.

##### 7.4.1.3 Statistical analysis limitations

No adjustment for multiple comparisons was applied despite conducting numerous correlation analyses across three groups (total, men, women) and multiple variables. This increases the probability of Type I errors (false positives). With approximately 30 correlation tests conducted, the expected number of spurious significant findings at *α* = 0.05 is 1–2 correlations.

The use of correlation coefficients, while appropriate for examining associations, provides limited information about the magnitude of relationships in practical terms. Correlation coefficients can be influenced by range restriction, outliers, and measurement error, potentially over- or underestimating true associations.

#### 7.4.2 Sampling and generalizability limitations

##### 7.4.2.1 Sample composition bias

The convenience sampling method and substantial gender imbalance (68% women vs. 32% men) severely limit generalizability. The Chilean older adult population exhibits approximately 55% women to 45% men (INE, 2021), indicating a 13-percentage-point overrepresentation of women in this sample. This imbalance has several consequences:

Statistical Power Implications: Post-hoc power analysis indicates that with *n* = 48 men, this study had approximately 60% power to detect medium effect sizes (r = 0.30) at α = 0.05, well below the conventional 80% threshold. This underpowering increases the probability of Type II errors (false negatives) in male subgroup analyses, making null findings difficult to interpret confidently.

Biased Prevalence Estimates: Gender-stratified prevalence estimates are imprecise due to small subsample sizes and non-representative sampling. Confidence intervals around prevalence estimates are wide, particularly for men.

Compromised Gender Comparisons: Direct statistical comparison of correlation coefficients between men and women was not conducted, preventing formal testing of whether observed gender differences in association strength are statistically significant. Visual differences in correlation magnitudes may reflect sampling variability rather than true population differences.

##### 7.4.2.2 Selection bias from hospital recruitment

Hospital-based recruitment introduces systematic selection bias. Participants accessing hospital services for the AudioBrain project likely differ from community-dwelling older adults who do not engage with healthcare systems in several ways:

- Higher health engagement and health literacy.
- Better functional status and mobility (able to travel to hospital).
- Potentially higher socioeconomic status (access to healthcare, transportation).
- Greater health concerns or symptoms prompting healthcare seeking.
- Exclusion of homebound or institutionalized individuals.

This selection may result in underestimation of social isolation and mental health problems, as the most isolated and depressed individuals may be less likely to participate in research studies or access hospital-based services.

##### 7.4.2.3 Geographic restriction

Geographic limitations to three urban municipalities within Santiago Metropolitan Region (Recoleta, Independencia, Santiago) prevents generalization to:

- Rural populations (approximately 12% of Chilean older adults), where social structures, family arrangements, and access to services differ substantially.
- Other regions of Chile with distinct cultural characteristics, socioeconomic profiles, and aging demographics.
- Small towns and cities, where community structures and social isolation patterns may differ from metropolitan Santiago.

Chile exhibits considerable geographic heterogeneity in aging experiences, with northern mining regions, southern agricultural areas, and coastal communities having distinct social and economic contexts that may influence loneliness and isolation patterns.

#### 7.4.3 Cultural, contextual, and unmeasured variables

##### 7.4.3.1 Cultural adaptation limitations

Beyond instrument validation concerns, the study lacked comprehensive cultural adaptation processes. No qualitative research explored how Chilean older adults conceptualize, experience, or express social isolation and loneliness. Cultural factors that may be particularly relevant but were not assessed include:

- Role of extended family networks versus friendship networks.
- Impact of traditional gender roles on social connection expectations.
- Community-level factors (neighborhood cohesion, social capital).
- Cultural attitudes toward aging and older adults’ roles in society.

##### 7.4.3.2 Unmeasured confounders and mediators

Several potentially important variables were not assessed, limiting understanding of mechanisms and confounding:

Socioeconomic factors: Income, education level, and financial strain can influence both social isolation and mental health. While municipalities were characterized by Social Priority Index, individual-level socioeconomic data were not collected.

Health status: Chronic health conditions, functional limitations, sensory impairments (hearing, vision), and cognitive function can all affect social participation and may confound associations between isolation and depression.

Social support quality: The study assessed social isolation frequency but not relationship quality, emotional support availability, or satisfaction with social connections—factors that may be more important than network size for mental health outcomes.

Life circumstances: Recent bereavement, retirement timing, living arrangements (living alone vs. with family), and caregiver status were not systematically assessed.

Pandemic exposure: Individual experiences during the pandemic (COVID-19 infection, loss of family members, economic impacts) may influence both current mental health and ongoing pandemic concerns but were not measured.

##### 7.4.3.3 Assessment timing limitations

Data collection occurred 2 years post-pandemic onset (2022), potentially missing the acute psychological impacts of initial lockdowns and social distancing measures. The assessment captured a period when many restrictions had been lifted, possibly resulting in:

- Underestimation of peak pandemic effects on social isolation and loneliness.
- Inability to examine trajectories of recovery or persistent difficulties.
- Confounding of pandemic-specific effects with general aging and life circumstances.

Recall bias may affect pandemic-related concern reports, as participants’ memories of pandemic experiences may have evolved over 2 years.

#### 7.4.4 Implications of limitations

These limitations collectively constrain the study’s conclusions in several important ways:

1. *Prevalence estimates* should be considered rough approximations rather than precise population parameters.
2. *Association strengths* may be under- or overestimated due to measurement error and sampling bias.
3. *Gender differences* require replication with adequate statistical power and representative sampling before drawing definitive conclusions.
4. *Generalizability* to broader Chilean older adult populations is uncertain and likely limited.
5. *Clinical recommendations* must remain tentative pending replication with more rigorous methodology.
6. *Causal interpretations* are inappropriate given the cross-sectional design.

Future research addressing these limitations—particularly through instrument validation, representative sampling, longitudinal designs, and comprehensive assessment of relevant confounders—is essential for advancing understanding of social isolation and loneliness in Chilean aging populations.

### 7.5 Future research directions

The limitations and findings of this study highlight several critical research priorities for advancing understanding of both social isolation and loneliness among Chilean and Latin American older adult populations. Given that these constructs demonstrated moderate correlation (*ρ* = 0.498) yet remained distinct phenomena with differential associations to mental health, future research must address both constructs systematically while examining their interrelationships.

#### 7.5.1 Immediate priorities: methodological foundations

The most urgent research priority is the cultural adaptation and validation of both social isolation and loneliness measures for Chilean and broader Latin American populations. This foundational work should include:

##### 7.5.1.1 Qualitative foundation

(a) Exploratory qualitative research examining how Chilean older adults conceptualize and distinguish between objective social disconnection (isolation) and subjective feelings of loneliness. (b) Investigation of cultural expressions of both constructs, including whether Spanish terminology (*aislamiento social, soledad*) captures the same constructs as English terms. (c) Examination of culturally-specific indicators of social isolation relevant to Chilean contexts (e.g., frequency of family gatherings, participation in *once/onces* tradition, community celebrations). (d) Understanding of cultural norms regarding acceptable levels of social contact and solitude versus problematic isolation.

##### 7.5.1.2 Psychometric validation

(a) Translation and cultural adaptation of the Steptoe Social Isolation Index, considering whether its five domains (unmarried/unpartnered, less than monthly contact with family/friends, no organizational participation) align with Chilean social structures. (b) Validation of the UCLA-3 Loneliness Scale or alternative loneliness measures, examining whether items function equivalently in Chilean populations. (c) Development of Chilean-normed cutoff scores for both social isolation and loneliness measures. (d) Examination of measurement invariance across gender, age groups, urban/rural locations, and socioeconomic strata to ensure measures function equivalently.

##### 7.5.1.3 Examining the isolation-loneliness relationship

(a) Investigation of discordant patterns: individuals high in isolation but low in loneliness (possibly representing chosen solitude or satisfying limited connections) versus those low in isolation but high in loneliness (large networks lacking emotional quality). (b) Identification of factors that moderate the isolation-loneliness relationship (personality, cultural values, relationship quality, living arrangements). (c) Exploration of whether the strength of association between isolation and loneliness varies across Chilean cultural contexts.

##### 7.5.1.4 Representative sampling and statistical power

Future studies must employ probability-based sampling methods to obtain population-representative data on both social isolation and loneliness:

National prevalence studies: (a) Nationally representative surveys establishing baseline prevalence of both social isolation and loneliness among Chilean older adults. (b) Stratified sampling ensuring adequate representation across gender, age, urban/rural location, regions, and socioeconomic levels. (c) Examination of co-occurrence patterns (proportion of older adult’s experience both isolation and loneliness; or Isolation without loneliness and vice versa).

Statistical power consideration: (a) Sample size calculations ensuring adequate power to detect associations for both isolation and loneliness with health outcomes. (b) Sufficient power for testing whether isolation and loneliness have independent effects on mental health when examined simultaneously. (c) Adequate sample sizes for gender-stratified analyses given this study’s underpowered male subsample (*n* = 48).

##### 7.5.1.5 Longitudinal and causal inference designs

Cross-sectional designs cannot establish whether social isolation leads to loneliness, loneliness leads to withdrawal and isolation, or bidirectional relationships exist. Priority research includes:

Prospective cohort studies: (a) Following older adults over multiple years to examine trajectories of both social isolation and loneliness. (b) Identifying critical transition points (retirement, widowhood, health decline) affecting both objective social networks and subjective loneliness. (c) Examining temporal sequences: Does increased isolation predict subsequent loneliness? Does loneliness lead to behavioral withdrawal and increased isolation?

Bidirectional relationships: (a) Cross-lagged panel models testing reciprocal effects between isolation and loneliness over time. (b) Investigation of whether isolation and loneliness have independent or synergistic effects on mental health deterioration. (c) Examination of factors that buffer or amplify the progression from isolation to loneliness (or vice versa).

#### 7.5.2 Expanded scope: understanding contextual factors

##### 7.5.2.1 Chilean and latin american cultural context

This study’s moderate correlation between isolation and loneliness (*ρ* = 0.498) may reflect cultural factors that deserve investigation: (a) Role of *familismo* in protecting against loneliness even when objective social isolation is high (due to intense emotional bonds with limited family contacts). (b) Impact of *personalismo* (preference for personal relationships) on whether large social networks without personal warmth lead to loneliness despite low isolation. (c) Cultural expectations about intergenerational co-residence and their violation leading to both isolation and loneliness. (d) Community-level factors (neighborhood cohesion, collective efficacy) that may prevent feelings of loneliness even when individuals have limited personal networks.

###### 7.5.2.1.1 Gender, isolation, and loneliness

This study found differential patterns by gender that require further investigation:

Women’s patterns: (a) Why did women show significant associations between both isolation and depression (*ρ* = 0.267) and loneliness and depression (ρ = 0.451)?; (b) Do women experience greater emotional distress from isolation because of socialized emphasis on relationship maintenance?; (c) Investigation of whether widowhood affects isolation and loneliness differently for women versus men.

Men’s patterns: (a) Why was no association found between isolation and depression among men (*ρ* = 0.014, *p* = 0.921)?; (b) Do men maintain adequate emotional connection despite objective isolation through different means?; (c) Exploration of whether masculine norms discourage acknowledgment of loneliness even when isolated. (d) Investigation of whether men’s social networks structured around activities buffer loneliness effects.

##### 7.5.2.2 Regional and geographic variations in both constructs

Urban–rural differences: (a) Whether rural populations experience high objective isolation but low loneliness due to tight-knit communities and cultural acceptance of geographic dispersion. (b) How urban anonymity may create loneliness without isolation (surrounded by people but lacking meaningful connection). (c) Impact of infrastructure (transportation, communication technology) on moderating isolation-loneliness relationships.

Regional comparisons: (a) Northern mining regions: Impact of male-dominated, transient work populations on community cohesion. (b) Central urban areas: Effects of urban sprawl and anonymity. (c) Southern rural areas: Role of agricultural traditions and community interdependence. (d) Coastal and island communities: Geographic isolation versus strong community bonds.

##### 7.5.2.3 Protective and risk factors for both isolation and loneliness

The moderate correlation between isolation and loneliness (*ρ* = 0.498) indicates that approximately 75% of variance is not shared, suggesting distinct protective and risk factors:

Factors specifically protecting against isolation: (a) Living arrangements (co-residence with family). (b) Functional mobility enabling community participation. (c) Access to transportation. (d) Participation in organizations (senior centers, religious groups). (e) Digital connectivity and technology skills.

Factors specifically protecting against Loneliness (Beyond network size): (a) Relationship quality and emotional intimacy. (b) Perceived social support availability. (c) Attachment security and social skills. (d) Cultural values emphasizing solitude versus connection. (e) Personality factors (introversion-extraversion, need for affiliation).

Risk factors affecting both: (a) Sensory impairments (hearing loss, vision problems) creating both communication barriers (isolation) and feeling disconnected (loneliness). (b) Cognitive decline affecting both social participation capacity and perception of connection. (c) Chronic health conditions limiting mobility (isolation) and creating feeling of being burdensome (loneliness). (d) Economic strain limiting social participation and creating social stigma.

Life course perspectives on isolation and loneliness: (a) Examination of how isolation and loneliness patterns change across old age (young-old 60–69, old-old 70–79, oldest-old 80+). (b) Investigation of cumulative effects: Does chronic isolation inevitably lead to loneliness over time?. (c) Cohort comparisons: How do current older adults’ experiences differ from future cohorts with different technological exposure and family structures?

##### 7.5.2.4 Pandemic-specific research on isolation versus loneliness

This study’s findings of persistent pandemic concern (91%) but minimal associations with either isolation or loneliness raise important questions:

Differential pandemic impacts: (a) Did pandemic restrictions increase objective isolation more than subjective loneliness due to normalized universal isolation?. (b) Investigation of whether technology-mediated connection during lockdowns prevented loneliness despite physical isolation. (c) Examination of long-term trajectories: Have isolation and loneliness levels returned to pre-pandemic baselines or remained elevated?

Pandemic concern relationships: (a) Why did pandemic concern show no associations with either isolation (*ρ* = −0.056) or loneliness (ρ = 0.028)?. (b) Investigation of whether pandemic concern represents distinct health anxiety rather than social concern. (c) The strong age-pandemic concern association among men (ρ = 0.456) deserves specific investigation regarding its independence from isolation and loneliness.

#### 7.5.3 Intervention and implementation research

##### 7.5.3.1 Evidence-based interventions for both constructs

Given that isolation and loneliness are related but distinct, interventions may need to target both simultaneously or address them through different mechanisms:

Isolation-focused interventions: (a) Programs increasing objective social contact opportunities (senior centers, group activities, transportation services). (b) Community infrastructure improvements facilitating social participation. (c) Technology training enabling digital connection. (d) Volunteer matching programs creating meaningful social roles.

Loneliness-focused interventions: (a) Cognitive-behavioral interventions addressing maladaptive social cognition. (b) Interventions improving relationship quality rather than quantity. (c) Befriending programs creating emotionally intimate connections. (d) Group interventions reducing stigma and normalizing loneliness experiences.

Integrated approaches: (a) Development of interventions simultaneously addressing both constructs. (b) Testing whether reducing isolation is sufficient to reduce loneliness or whether direct loneliness intervention is required. (c) Examination of optimal sequencing: Should isolation be addressed before loneliness interventions, or simultaneously?

Gender-specific intervention approaches: Given differential associations observed (women: stronger loneliness-depression link; men: no isolation-depression link), research should examine:

For Women: (a) Interventions addressing loneliness and relationship quality given stronger loneliness-depression associations. (b) Programs supporting women through major transitions (widowhood) affecting both isolation and loneliness.

For Men: (a) Strategies engaging men who may be isolated but not reporting loneliness or depression. (b) Activity-based programs reducing isolation while minimizing emphasis on emotional disclosure. (c) Investigation of whether men benefit more from objective network enhancement versus loneliness-focused cognitive interventions.

Evaluation of Existing Chilean Programs: (a) Assessment of whether SENAMA programs (Vínculos, Buen Vivir) reduce isolation, loneliness, or both - Identification of program components most effective for each construct. (b) Cost-effectiveness analyses comparing isolation-focused versus loneliness-focused interventions.

##### 7.5.3.2 Healthcare system integration for both constructs

Screening Protocols: (a) Development of brief screening protocols assessing both isolation and loneliness in primary care. (b) Investigation of whether screening both constructs provides added value over screening only one. (c) Establishment of referral pathways differentiated by whether patients experience isolation, loneliness, or both.

Social Prescribing Adaptations: (a) Matching social prescriptions to whether individuals experience isolation (need for connection opportunities) versus loneliness (need for relationship quality improvement). (b) Training link workers to distinguish isolation from loneliness and prescribe appropriately. (c) Evaluation of whether addressing objective isolation through social prescriptions reduces subjective loneliness.

#### 7.5.4 Methodological innovations

##### 7.5.4.1 Advanced statistical approaches for both constructs

Network analysis: (a) Examination of complex interrelationships among isolation, loneliness, depression, anxiety, health conditions, and functional limitations. (b) Investigation of central nodes in causal networks: Which constructs are most influential?.

Latent class/profile analysis: (a) Identification of subgroups with distinct patterns: high isolation/high loneliness, high isolation/low loneliness, low isolation/high loneliness, low isolation/low loneliness. (b) Examination of whether these profiles have different demographic characteristics, risk factors, and mental health outcomes. (c) Development of profile-specific interventions.

Mediation and Moderation Analyses: (a) Testing whether loneliness mediates relationships between isolation and mental health. (b) Identifying moderators that strengthen or weaken isolation-loneliness associations. (c) Examination of cultural values as moderators.

Machine Learning Approaches: (a) Predictive modeling identifying individuals at high risk for progression from isolation to loneliness. (b) Identification of protective factors distinguishing those who remain resilient despite isolation.

#### 7.5.5 Policy-relevant research

##### 7.5.5.1 Economic analysis for both constructs

Healthcare Cost Studies: (a) Separate and combined healthcare costs attributable to isolation versus loneliness. (b) Investigation of whether costs differ: Does loneliness have greater mental health costs while isolation has greater physical health costs?

Cost-Effectiveness of Interventions: (a) Comparative cost-effectiveness of isolation-focused versus loneliness-focused interventions. (b) Analysis of whether preventing isolation is more cost-effective than treating established loneliness.

Policy Evaluation: Age-Friendly Cities Impact: (a) Evaluation of whether age-friendly infrastructure reduces isolation, loneliness, or both. (b) Assessment of differential benefits by urban versus rural implementation.

Social Policy Impacts: (a) Examination of how pension adequacy affects both isolation (ability to afford social participation) and loneliness (financial stress affecting relationship quality). (b) Investigation of housing policies’ impacts on both constructs (e.g., intergenerational housing, co-housing models).

Health Equity: (a) Examination of whether poverty affects isolation and loneliness through the same or different mechanisms. (b) Investigation of whether interventions are equally effective across socioeconomic levels for both constructs.

Marginalized Populations: (a) Assessment of isolation and loneliness among indigenous older adults, LGBTQ+ elders, and immigrant populations. (b) Investigation of whether cultural minority status affects isolation and loneliness differently.

#### 7.5.6 Regional collaboration and integrated research agenda

##### 7.5.6.1 Latin American research networks

(a) Harmonized measurement of both social isolation and loneliness across Latin American countries. (b) Comparative research examining whether cultural variations affect isolation-loneliness relationships differently across countries. (c) Cross-national replication of this study with validated instruments and improved methodology.

##### 7.5.6.2 Interdisciplinary integration

Given that isolation is often studied by sociologists and epidemiologists while loneliness is primarily studied by psychologists, integrated approaches are needed: (a) Collaboration across disciplines ensuring both constructs receive equal attention. (b) Development of theoretical models explicitly incorporating both isolation and loneliness as distinct but related constructs. (c) Training programs educating researchers about both constructs and their interrelationships.

In summary, future research must systematically address both social isolation and loneliness as distinct constructs while examining their interrelationships and differential impacts on mental health among Chilean older adults. The moderate correlation observed (*ρ* = 0.498) indicates these are related but not redundant constructs, requiring parallel research attention. Only through comprehensive research addressing both phenomena can effective, targeted interventions and policies be developed.

## 8 Conclusion

This study represents one of the first systematic examinations of social isolation, loneliness, and their associations with depressive symptoms among Chilean older adults in the post-pandemic period. Despite significant methodological limitations, the findings provide important preliminary insights into these understudied phenomena in a Latin American context and establish a foundation for future research.

### 8.1 Key findings and contributions

The study documented concerning prevalence rates: 42% of participants presented social isolation, 26% reported loneliness, and 30% exhibited depressive symptoms. These estimates, while requiring cautious interpretation due to convenience sampling and hospital-based recruitment, align with elevated mental health concerns observed during Chile’s pandemic period and suggest that psychosocial challenges persist even after acute pandemic restrictions have ended.

The moderate correlation between social isolation and loneliness (*ρ* = 0.498, *p* < 0.001) provides Chilean evidence supporting the theoretical distinction between objective social disconnection and subjective feelings of loneliness established in international literature. This finding is particularly important given the absence of prior Chilean studies examining these constructs simultaneously with validated measurement approaches.

Gender-specific patterns emerged, with women demonstrating stronger associations between loneliness and depressive symptoms (ρ = 0.451, *p* < 0.001) compared to men (ρ = 0.223, *p* = 0.126), though the underpowered male subsample prevents definitive conclusions. This pattern suggests potential gender differences in vulnerability to loneliness-related mental health impacts that warrant further investigation in adequately powered studies.

Notably, the study found minimal associations between pandemic-related concerns and either social isolation or loneliness, despite 91% of participants reporting high levels of persistent pandemic worry 2 years post-pandemic onset. This unexpected finding suggests that health-related anxieties may operate independently from social connectedness concerns, though the exceptionally high prevalence of enduring pandemic concern indicates psychological impacts requiring continued attention.

### 8.2 Methodological limitations and their implications

Several critical methodological limitations significantly constrain the generalizability and clinical applicability of these findings. The use of instruments not validated in Chilean populations (Steptoe Social Isolation Index, UCLA-3 Loneliness Scale) represents a fundamental limitation, as cultural differences in conceptualizing and expressing social connection may affect measurement accuracy. The convenience sampling method, substantial gender imbalance (68% women), and hospital-based recruitment introduce systematic biases that limit generalizability to the broader Chilean older adult population. The cross-sectional design precludes causal inferences about relationships between variables.

These limitations are not unique to this study but reflect broader challenges facing aging research in Latin American contexts, where validated instruments, research infrastructure, and funding for population-representative studies remain limited. Acknowledging these limitations is essential for appropriately contextualizing findings and guiding future research priorities.

### 8.3 Value and positioning

Despite methodological constraints, this study makes several important contributions:

Regional context: It adds to the limited body of research on social isolation and loneliness among Latin American older adults, providing Chilean data that can inform regional comparisons and highlight shared challenges across Latin American countries experiencing rapid demographic aging.

Post-pandemic assessment: The timing of assessment (2 years post-pandemic onset) captures a period of transition that remains understudied, filling a gap between acute pandemic research and long-term impact studies.

Dual construct examination: By examining both social isolation and loneliness simultaneously, the study demonstrates their interrelationship while supporting their conceptual distinction, providing empirical basis for interventions targeting both constructs.

Gender considerations: The gender-stratified analyses, though limited by sample size, highlight potential sex differences that require further investigation in Chilean contexts.

Baseline establishment: This research establishes preliminary baseline data that can inform future hypothesis generation, sample size calculations, and study design for more rigorous investigations.

### 8.4 Immediate implications despite limitations

While definitive clinical recommendations and policy prescriptions are premature given methodological limitations, several tentative implications emerge:

Healthcare system: The high prevalence of co-occurring social isolation, loneliness, and depressive symptoms suggests potential value in integrated screening approaches within Chile’s primary healthcare system, pending validation of appropriate instruments.

Research priorities: The findings underscore the urgent need for cultural adaptation and validation of social isolation and loneliness measures in Chilean populations as a foundational step for advancing this research area.

Program development: Existing community programs (e.g., SENAMA initiatives) should be rigorously evaluated using validated measures to establish their effectiveness in reducing isolation and loneliness before widespread scaling.

Gender sensitivity: The observed gender differences, though requiring replication, suggest that gender-sensitive approaches to assessment and intervention design warrant consideration.

### 8.5 Critical next steps

This study’s findings and limitations collectively point to several critical research priorities:

Immediate Priorities (Years 1–2): (a) Cultural adaptation and psychometric validation of social isolation and loneliness measures for Chilean populations. (b) Qualitative research exploring how Chilean older adults conceptualize and experience these constructs. (c) Development of sampling frameworks enabling probability-based recruitment for future studies.

Medium-Term Priorities (Years 3–5): (a) Population-representative prevalence studies using validated instruments to establish accurate baseline estimates. (b) Longitudinal cohort studies examining trajectories and causal relationships. (c) Rigorous evaluation of existing intervention programs using standardized outcome measures.

Long-Term Priorities (Years 5–10): (a) Development and testing of culturally adapted interventions. (b) Implementation of research establishing feasible approaches for healthcare system integration. (c) Policy evaluation research examining impacts of age-friendly cities, social prescribing, and other initiatives.

Latin American Collaboration: (a) Establishment of regional research networks to harmonize measurement, share resources, and enable cross-national comparisons. (b) Collaborative development of culturally appropriate assessment tools applicable across Latin American contexts.

### 8.6 Final perspective

This study should be interpreted as an exploratory investigation that identifies important patterns and relationships while simultaneously highlighting substantial methodological gaps requiring attention. The results are not definitive but rather hypothesis-generating, establishing preliminary evidence that warrant replication using more rigorous methodological approaches before informing clinical practice or policy decisions.

The documented prevalence of social isolation (42%) and loneliness (26%), if confirmed in representative samples, would represent a significant public health challenge for Chile’s rapidly aging population. The associations observed between these constructs and depressive symptoms, though requiring causal verification through longitudinal research, suggest potential intervention targets for improving mental health outcomes among Chilean older adults.

Importantly, the methodological limitations identified in this study reflect broader challenges facing aging research in Latin American contexts. Addressing these challenges requires sustained investment in research infrastructure, instrument validation, researcher training, and establishment of longitudinal cohorts. Only through such comprehensive efforts can the evidence base necessary for developing effective, culturally appropriate interventions and policies be established.

The high prevalence of persistent pandemic-related concerns (91%) 2 years after acute pandemic restrictions ended is perhaps the study’s most unexpected finding, suggesting psychological impacts that extend well beyond the immediate crisis period. Whether these concerns represent adaptive vigilance, maladaptive anxiety, or rational assessment of ongoing health threats requires further investigation but highlights the enduring psychological toll of the pandemic on older adults.

In conclusion, while this study’s methodological limitations preclude definitive conclusions, it provides valuable preliminary evidence establishing the relevance of social isolation and loneliness as mental health concerns among Chilean older adults. The findings underscore the urgent need for methodologically rigorous research employing validated measures, representative sampling, and longitudinal designs to advance understanding of these critical public health challenges in Chilean and broader Latin American aging populations. Only through such systematic research efforts can evidence-based interventions and policies be developed to promote social connectedness and mental health among rapidly growing populations of older adults in the region.

## Data Availability

The datasets presented in this article are not readily available as they are restricted due to: (1) ethical approval requirements, (2) participant privacy protections, (3) institutional data sharing policies, and (4) Chilean data protection regulations. Data can be made available to qualified researchers upon reasonable request with appropriate ethical approvals. Requests to access the datasets should be directed to christinegierke@uchile.cl.
